# Development and application of an aggregate adherence metric derived from population pharmacokinetics to inform clinical trial enrichment

**DOI:** 10.1007/s10928-015-9414-4

**Published:** 2015-03-29

**Authors:** Jonathan Knights, Shashank Rohatagi

**Affiliations:** Otsuka Pharmaceutical Development & Commercialization, Inc., 508 Carnegie Center Blvd. Suite 300, Princeton, NJ 08540 USA

**Keywords:** Aggregate adherence metric, Population pharmacokinetics, Steady-state plasma drug concentrations, Simulation, Aripiprazole

## Abstract

Nonadherence to prescribed medication is a common barrier to effective treatment, and current options to determine adherence are limited. This study describes development of an aggregate adherence measure based on population pharmacokinetics (PK), and its comparison to a subjective questionnaire, the Morisky 8-item medication adherence scale (MMAS8), in a trial of psychiatric patients on stable doses of oral aripiprazole. A comprehensive model was first built using plasma drug concentration data from 24 clinical studies comprising 448 patients with over 13,500 observations. Application of this model to independent patient profiles for a given drug-dosing regimen were used to generate the primary aggregate adherence metric, a ratio of observed versus expected plasma exposures at steady-state. Although the metric is capable of comparing relative adherence across groups, simulations showed that the metric is not sufficiently sensitive as an individual diagnostic in all cases. There were no trends observed between results from calculated aggregate adherence metrics and total scores from MMAS8 in a single-visit clinical trial of 47 patients with bipolar 1 disorder or schizophrenia who were on stable doses of aripiprazole, although a strong association was observed for one MMAS8 question. The range of the metric calculated for patients was between 0.16 and 3.15. The described approach of a novel “reverse” application of population PK to quantify relative adherence with an aggregate measure may be influential for both clinical and pharmacometric communities.

**Electronic supplementary material:**

The online version of this article (doi:10.1007/s10928-015-9414-4) contains supplementary material, which is available to authorized users.

## Introduction

There is currently great interest in the pharmaceutical and clinical literature regarding nonadherence to a prescribed medication [1–5]. Nonadherence is highly prevalent and remains a major barrier to achieving optimal health outcomes. In psychotic disorders in particular, a conservative estimate for nonadherence to prescribed medication is 50 % based on current literature [6]. Additionally, in psychiatry, poor adherence has been associated with symptom exacerbation [7, 8], relapse [9–12], rehospitalization [13] and an increased risk of suicide [14–16]. Despite the well-replicated association between adverse clinical outcomes and sub-optimal medication adherence, deviation from medication adherence is difficult to detect and current methods to determine adherence have substantial limitations. These various methods and their respective limitations have been extensively reviewed elsewhere [17].

Previous efforts handling medication adherence have focused on maximizing the accuracy of ingestion records for analyses [18–20], or describing observed adherence patterns derived from electronic monitoring systems [21], pharmacy claims [10], or other methods. In the present work, the goal was to specifically quantify a measure of relative adherence across psychiatric patients, and, subsequently, use sparse plasma sampling to evaluate whether or not subjective results from a clinical questionnaire could serve as a surrogate measure for adherence levels. The Morisky 8-item medication adherence scale (MMAS8), previously validated for hypertension patients [13], was used for the subjective questionnaire, whereas steady-state plasma drug exposure was selected as the objective measure. Our goal was to assess correlation between the subjective measure, purported to detect medication nonadherence, and the objective measure of relative expected steady-state exposures of aripiprazole. We wished to assess whether or not suboptimal, subjectively measured, adherence behavior would be associated with lower steady-state plasma drug exposures.

To construct an aggregate adherence metric, a novel ‘reverse’ application of population pharmacokinetics (POPPK) was applied to oral aripiprazole treatment. In a standard POPPK analysis, doses are administered and recorded, and plasma sampling over time is used as the basis for the development of structural and statistical models. In our ‘reverse’ application of POPPK, we started by building a PK model from historical clinical trial data, then drew plasma samples from an independent population, and finally used model outputs to generate a metric of observed versus expected exposures, driven by average adherence levels. The analysis was conducted in multiple stages. First, a comprehensive population PK model of oral aripiprazole was built with 24 studies submitted as part of the original new drug application [22]. The PK model was to be fixed for the purpose of estimating independent patient profiles for given prescribed dosing regimens, and a simple ratio of observed versus expected exposures at steady-state was adopted as the primary metric for comparison. Second, simulations were conducted to assess the extent to which the calculated metric might be interpretable at the individual level (as opposed to the group level). Finally, MMAS8 scores and plasma blood concentrations of aripiprazole were measured during a clinical trial and evaluated for comparison using the developed population PK model. The work stream and results from this analysis may be influential for the clinical and pharmacometric communities alike; however, it is the “reverse” application of population PK to quantify relative adherence across groups that is truly unique.

## Methods

### Population PK model

**Fig. 1:**
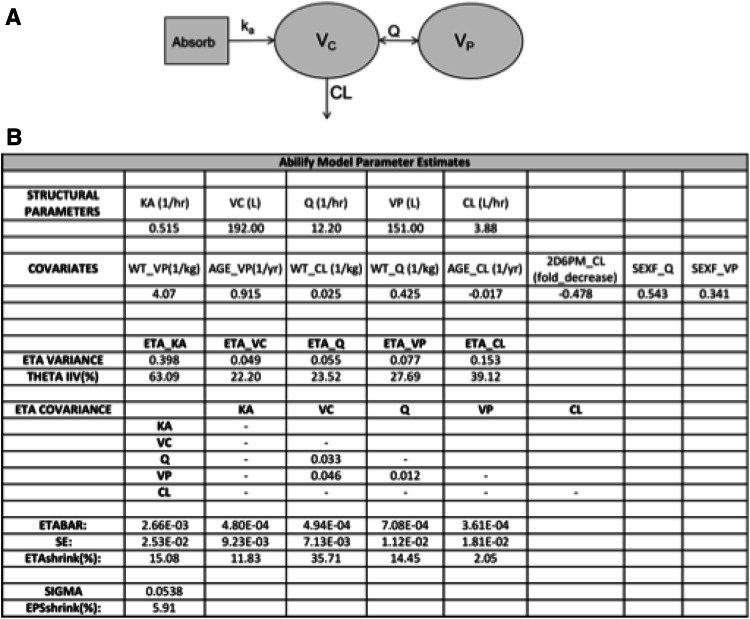
Summary for the population PK model developed for oral aripiprazole. **a** Example of 2-compartment structural model used to describe aripiprazole PK. Model parameters include the first-order absorption rate constant (k_a_), the theoretical volume of distribution of the central compartment (V_C_), the first-order inter-compartmental transfer rate (Q), the apparent clearance of the drug from the central compartment (CL), and the theoretical volume of distribution of the peripheral compartment (V_P_). **b** Final model parameter estimates with the following covariate values: the effect of weight on V_P_ (WT_V_P_), CL (WT_CL), and Q (WT_Q), the effect of age on V_P_ (AGE_V_P_), CL (AGE_CL), and the effect of CYP2D6 poor metabolizer phenotype on CL (2D6PM_CL)

A POPPK model was developed for oral administration of aripiprazole using data from 24 clinical studies comprising 448 individuals with over 13,500 observations. A 2-compartment model with first-order absorption was found to best describe the data. Interindividual variability terms were present on all structural model parameters, and a proportional residual error model was used. Additionally, three off-diagonal covariance elements were included between the volume of the central compartment (V_C_), the intercompartmental transfer rate (Q), and the volume of the peripheral compartment (V_P_, Fig. 1). The heterogeneous nature of data collection and inconsistent availability of covariates across studies inherently manifested in some modeling limitations. The ultimate intention of the modeling effort was to capture a robust upper-bound of the true magnitude of variability in the general population for the structural model parameter values. Therefore, it was preferred to include all data and accept a larger unexplained variability on the population parameters, rather than to select a subset of studies with comprehensive covariate information and reduce the unexplained variability. Additionally, it is believed that any improvement in explained variability would only strengthen the application of the model application that is discussed in the following sections. It is assumed that adherence in a clinical trial setting provides an upper boundary for adherence levels in the real world (e.g., white-coat phenomenon). It is also assumed that the presence of any patient reporting error in dosing times increases the magnitude of observed variability in the population and (along with the large sample size) is in line with the objective of providing an upper-bound of the true magnitude of variability on the parameters.

**Fig. 2:**
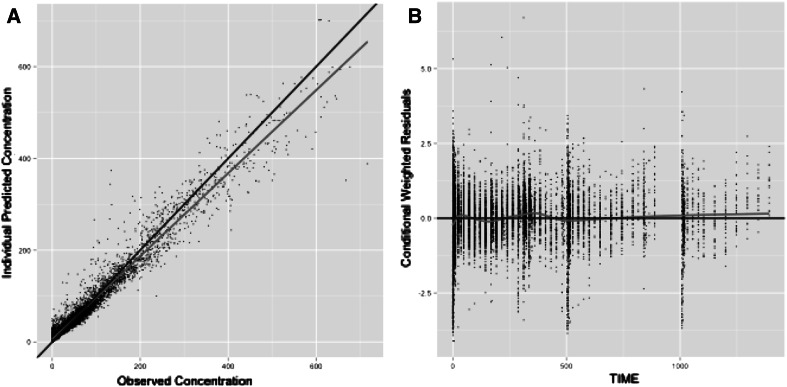
Diagnostic plots for the aripiprazole population PK model described in Fig. 1**a** predicted versus observed concentrations. The *blue line* represents the line of identity; the *red line* represents the best fit for the data and covers only the range of observed data points. **b** A plot of the conditional weighted residuals versus time. The overlaid fitted line is a Loess plot (Color figure online)

This article focuses on describing a novel application of POPPK, and therefore it does not show the full details of pharmacometric model construction. Nonetheless, brief model diagnostics and parameter estimates are provided (Figs. 1, 2), and the final model equations are present in the supplementary material. Although the highest observed concentrations tended to be underpredicted (which is not uncommon for oral medications without intravenous data), model performance was adequate: visual predictive plots of observed versus predicted concentrations for the 24 studies used in building the model are presented as supplementary material.

### Simulations

To assess whether or not the aggregate adherence metric could be used as an individual diagnostic, in addition to its primary purpose of comparison across groups, 31 days of possible dosing events were simulated for 5000 subjects equally assigned to either an adherent or a nonadherent group. Subjects in the adherent group were assigned an 80 % probability of taking each scheduled dose, while patients in the nonadherent group were assigned a 40 % probability of taking each scheduled dose. Whether a dose was taken for each subject was decided using independent Bernoulli calls with success rate consistent with the probability of a dosing event given the subject’s adherence group. Four concentration time points were simulated for each subject at the end of the 31 days to closely follow the design of a subsequent clinical study: concentrations were simulated at 8, 9, 10, and 11 h after the dosing event. As performance at the individual level is not the primary focus of this work, this simplified version of adherence presents a baseline assessment. It is assumed that performance of the aggregate adherence metric at the individual level would be reduced with more complicated models of adherence.

Three sets of simulations were conducted to test the performance of the adherence metric under constant population parameters (thetas) and different variance conditions: (1) the original model unchanged, (2) the coefficient of variation (CV) of the apparent clearance term decreased by half, and (3) all interindividual variability terms reduced by half, with the relevant covariance terms reduced by one fourth as Cov(*a* ***X***,*b* ***Y***) = *ab*Cov(***X***,***Y***), where both *a* and *b* are equal to 0.5 and ***X*** and ***Y*** are separate eta terms. Covariate values for the simulation dataset were resampled as a block (with replacement) from the original model-building data set. This was done to ensure maintenance of the covariance structure expected from the model.

Using the simulated individual predicted (IPRED) concentrations, data sets were generated to estimate individual parameter values under the assumption that all dosing events occurred (e.g., 100 % adherent). All model parameter estimates were fixed with the exception of the interindividual variability term for apparent clearance (CL_app_) and the residual variability. Given the sparse sampling nature of the profiles, a three-phase sequential estimation methodology was used (designed to mimic what would be used for application to the clinical data as explained below); simulation and estimation control stream examples are provided as supplementary materials.

### Aggregate adherence metric

Upon completion of parameter estimation under the assumption of full adherence, an aggregate adherence metric was calculated for each subject. Given that all values of model parameters were fixed to prior estimates (with the exception of the interindividual variability term on CL_app_ and residual error term), it may be asserted that significant deviations from the expected value—after accounting for relevant covariate values—are partially attributable to nonadherence (or over-adherence, i.e., taking too much medication). The underlying idea is that systematic nonadherence contributes to deviations in expected drug exposures at steady-state (AUC_ss_), which may be observed and at least partially quantified. The following metric was calculated for each subject:

1 $$ADHMET = \frac{{AUC_{SS,obs} }}{{AUC_{SS,\exp } }} = {\raise0.7ex\hbox{${\frac{Dose}{{\tau \times CL_{obs} }}}$} \!\mathord{\left/ {\vphantom {{\frac{Dose}{{\tau \times CL_{obs} }}} {\frac{Dose}{{\tau \times CL_{\exp } }}}}}\right.\kern-0pt} \!\lower0.7ex\hbox{${\frac{Dose}{{\tau \times CL_{\exp } }}}$}} = \frac{{CL_{\exp } }}{{CL_{obs} }}$$

*AUC*_*SS,obs*_ is the observed area under the concentration–time curve at steady-state, based on the Bayesian post hoc estimate of clearance following the estimation procedure in NONMEM; *AUC*_*SS,exp*_ is derived from the expected value for clearance given a subject’s covariate values from the final POPPK model; and *τ* is the dosing interval (24 h). The final equation for the expected value of clearance in the model was:

2 $$CL_{\exp } ({\raise0.7ex\hbox{$L$} \!\mathord{\left/ {\vphantom {L {hr}}}\right.\kern-0pt} \!\lower0.7ex\hbox{${hr}$}}) = TVCL = \left[ {3.88 + WTA*0.0251*\;\left( {WT - 74.19} \right) - 0.0167*(AGE - 32)} \right]*(1 - 0.478*2D6PM)$$

WTA is a binary variable indicating subject’s weight ≤115 kg (0 = no, 1 = yes) and 2D6PM is a binary variable indicating subject’s CYP2D6 poor metabolizer phenotype (0 = no, 1 = yes). CYP2D6 genotype is a well-documented covariate for aripiprazole metabolism, and the remaining covariates in this model are similar to those found in previous internal reports (data not published). The final observed CV for the apparent clearance parameter in the developed model was ~39 %, indicating that fully adherent subjects may generate adherence metrics, as described above, between ~60 and 140 %. Consequently, our initial test decision boundary for a nonadherent subject via the aggregate adherence metric (Eq. 1) was assigned to <60 % for the simulations testing utility at the individual level.

### Clinical data

The clinical trial conducted for this analysis enrolled 47 patients (31 male) 18–55 years of age, with a current diagnosis of bipolar 1 disorder (n = 15) or schizophrenia (n = 32) (*Diagnostic and Statistical Manual of Mental Disorders, Fourth Edition, Text Revision [DSM*-*IV*-*TR]* criteria, NCT02050854). Patients had been treated with oral aripiprazole (10, 15, 20, or 30 mg) for at least 2 weeks before blood sample collection in order to presume steady-state. Blood samples for measurement of aripiprazole plasma concentrations were collected from eligible patients at visit arrival (hour 0) and at 1, 2, and 3 h post-arrival. Patients reported the date and time of the last taken dose of aripiprazole, and completed the MMAS8. Aggregate adherence metric (Eq. 1) values were plotted against total MMAS8 scores and also against responses to individual MMAS8 questions. The study was conducted in accordance with International Conference on Harmonisation Good Clinical Practice guidelines for conducting, recording, and reporting trials, as well as for archiving essential documents. Consistent with ethical principles for the protection of human research subjects, no procedures were performed before study candidates had signed the informed consent form (ICF). The ICF, protocol, and amendments for this trial were approved by the institutional review board or independent ethics committee.

### Hardware and software

Data manipulation and data set creation were performed with user-written code in the Java programming language within the NetBeans8.0 integrated development environment. NONMEM 7.2 was used for PK model building, simulation, and estimation. Graphics were constructed in the R statistical programming language (v.3.0.3).

## Results

### Simulations

**Fig. 3:**
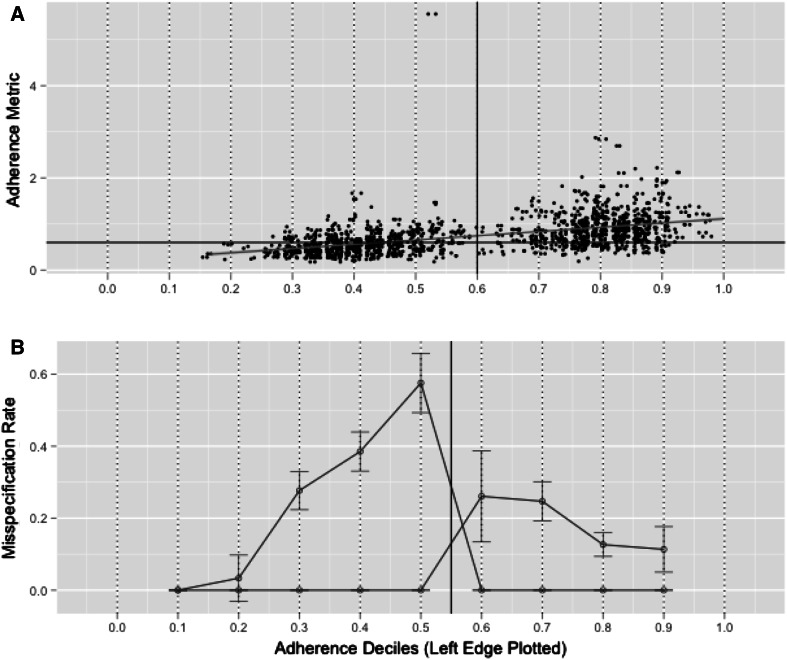
Results from simulations to assess the relationship of the calculated adherence metric to the true simulated adherence. **a** Observed adherence metric, calculated with Eq. 1 versus the true simulated adherence. The *solid reference lines* represent the 0.6 adherence decision boundary specified from the original model. A best-fit linear regression line is overlaid. **b** Misspecification rate of the calculated adherence metric with respect to the 0.6 adherence decision boundary (*vertical reference line*) within each decile of adherence. The misspecification rate (*open circles*) are plotted on the left boundary of the respective decile group, that is, the misspecification rate of those patients who were between 20 and 30 % is represented by the *open circle* at *x* = 0.2. The *error bars* represent the calculated standard error of the proportion. Two separate lines are plotted for the misspecification of nonadherent patients and adherent patients. The misspecification error for the nonadherent patients is > 0 to the left of the 0.6 reference line, and 0 to the right of the vertical reference line. Conversely, the misspecification error for the adherent patient group is > 0 to the right of the 0.6 reference line, and 0 to the left of the vertical reference line

**Fig. 4:**
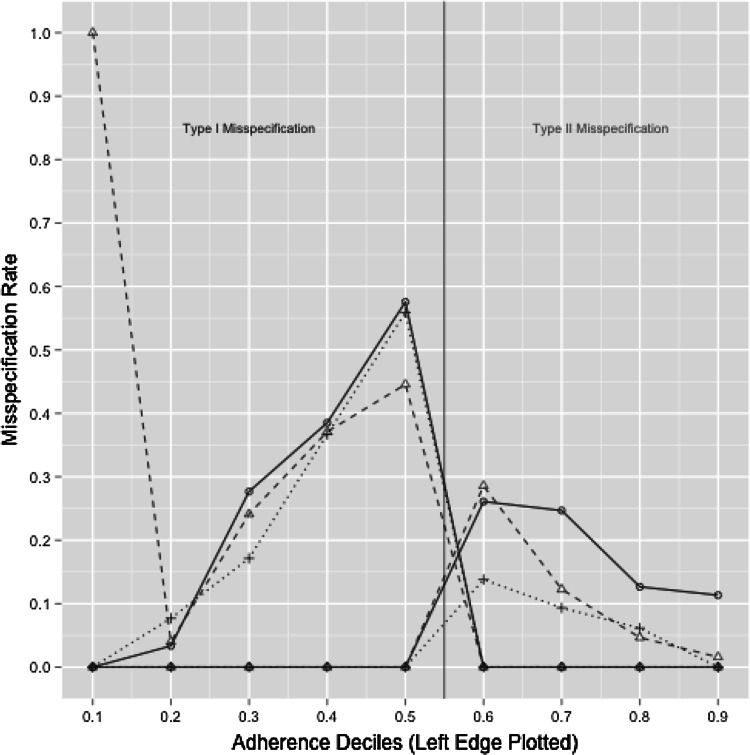
Adherence metric results from simulations to assess the performance of the adherence metric under different variance parameter values within the population PK model. Two separate lines are plotted for each simulation. Misspecification error of patients in the nonadherent group is presented to the left of the vertical reference line at *x* = 0.55 under the “Type I Misspecification” heading, similar to a type I (alpha) error rate, similar to accepting the null hypothesis when a true difference exists. Likewise, the “Type II Misspecification” is analogous to a type II (beta) error rate, similar to rejecting the null hypothesis when no true difference exists. The misspecification error for the nonadherent patients is > 0 to the left of the* x* = 0.55 reference line, and 0 to the right of the vertical reference line. Conversely, the misspecification error for the adherent patient group is > 0 to the right of the 0.6 reference line, and 0 to the left of the vertical reference line. The results from the model with all variance parameters being kept the same are shown as the *solid blue line with open circles*; the results of the model with only the CV of the apparent clearance parameter decreased by half are shown as the *dashed green line* *with open triangles*; and the results of the model with all parameter CVs decreased by half are shown as the *dotted line* *with plus symbols* (Color figure online)

Figure 3 shows the relationship between the aggregate adherence metric and the true underlying adherence at the individual level from simulations (Fig. 3a), as well as the misspecification rate of the adherence metric (±SE) at the individual level, with respect to observed deciles of the true adherence (Fig. 3b). Using a value of 0.6 as the adherence decision boundary, the misspecification rate for classifying a patient as adherent given the current study design was much lower than for classifying a patient as nonadherent. “Type I Misspecification” indicates that the error is similar to a type I (alpha) error rate, equivalent to accepting the null hypothesis when a true difference exists. Likewise, the “Type II Misspecification” is analogous to a type II (beta) error rate, similar to rejecting the null hypothesis when no true difference exists. The crossover pattern around 0.55 in Figs. 3 and 4 represents the switch from nonadherent misspecification to adherent misspecification based on the 60 % decision boundary. In the closest deciles near the decision boundary (50–60 and 60–70 %), where performance is expected to be the least sensitive, the difference in misspecification rate for classifying a patient as adherent versus nonadherent was approximately twofold (26.1 vs. 57.6 %, respectively). However, the misspecification rate was below 20 % for patients who were either <30 % adherent or patients who were ≥80 % adherent.

Misspecification rates from additional simulations changing the degree of variance in the model parameters are displayed in Fig. 4. For clarity, the standard errors are not included as the intent is to show relative effect of altering the variance structure rather than statistical significance. For these simulations, the decision boundary was kept constant at 0.6. When the CV of the clearance parameter was reduced from ~40 to 20 %, there was an improvement in type I misspecification rate (Fig. 4, dashed line). When all CV terms were reduced by half (including covariance terms), there was no apparent improvement in type I misspecification of nonadherent behavior, but there was a marked decrease in misspecification of adherent behavior (Fig. 4, dotted line). Separate comparisons were made after moving the decision boundary to 0.8 for simulations with 20 % CV on the clearance parameter (not shown). Under these conditions, the misspecification rate around the decision boundary was approximately 50 and 40 % for type I and type II misspecification, respectively. The increase in type-II misspecification with decreased IIV and a decision boundary set at (1 − CV_CL_)/100 suggests that the true underlying adherence levels are also contributing to the type-II misspecification rates; in other words, a highly adherent population and a model with low IIV will still yield a relatively high type-II error rate near the decision boundary. Provided the decision boundary is not near the upper limit of adherence for the population, the results demonstrate that the aggregate adherence metric may serve as an indicator of adherence or nonadherence in cases where the observed metric is well outside the expected variability range for the clearance parameter.

This simplified scenario of adherence simulations demonstrate that *ADHMET* values are not appropriate for individual level diagnostics; however, utility as an individual diagnostic is unrelated to its appropriateness as a measure of relative adherence within groups, and its ability to serve as the basis for a statistical comparison across groups under presumed conditions of homogenous variance.

### Clinical PK results

**Fig. 5:**
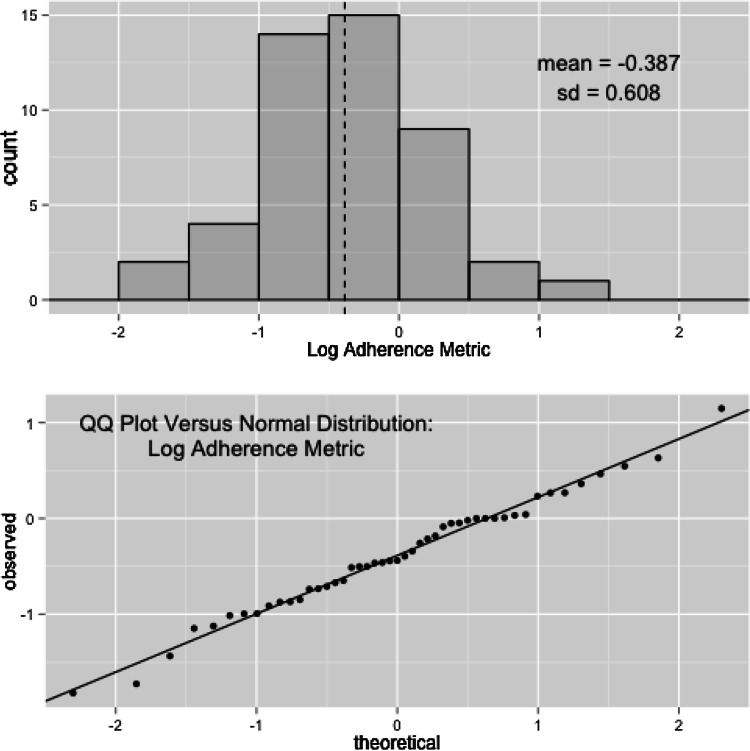
Distribution for calculated adherence metric values in the clinical study population

 Summary statistics of the aggregate adherence metric for the 47 patients evaluated in this study are provided in Table 1. The average (mean) adherence metric calculated for the clinical data (back calculated from the log-domain) was 0.679, with a median value of 0.644, and a range of 0.163–3.131. Figure 5 shows the distribution of the logarithm of the adherence metric in the population with the corresponding QQ-plot comparing the distribution to a hypothetical normal distribution. Interestingly, there was a significant difference in *ADHMET* values across gender in this study (see supplementary material); however, this relationship is not explored in more detail as the primary comparison is based solely on questionnaire responses, and their ability to serve as surrogates for nonadherent behavior *a priori*.

**Table 1:** Summary statistics for aggregate adherence metric calculated from clinical data

N	Mean	Median	Range
Descriptives in log domain			
47	−0.387	−0.44	−1.83–1.148
Descriptives back-calculated to linear domain			
47	0.679	0.644	0.163–3.151
Descriptives expressed as percent (%)			
–	67.9	64.4	16.2–315.1

### Clinical questionnaire correlations

**Fig. 6:**
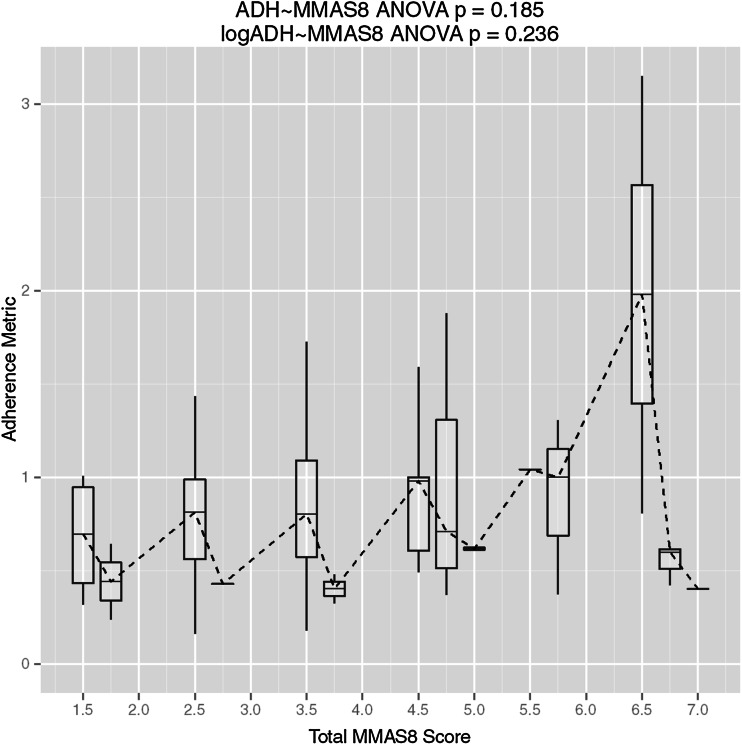
Box plots for the distribution of calculated adherence metrics within each group scoring a particular value on the MMAS8 with the corresponding analysis of variance analysis (ANOVA) *P* value testing for a difference across groups for both the ordinary and log domains. The *dashed line* connects the median values within each group

**Fig. 7:**
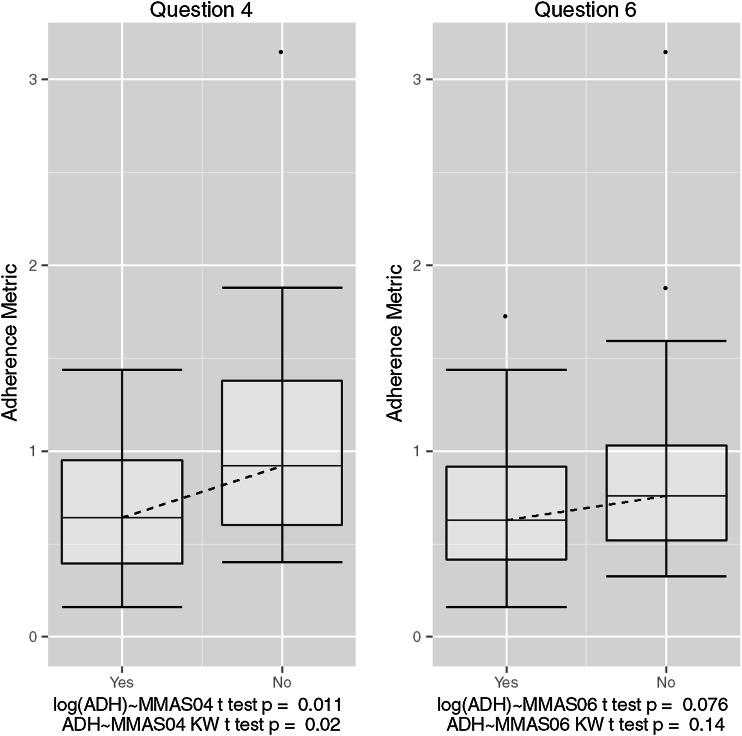
Box plots for the distribution of calculated adherence metric within each response group for questions 4 (*left*) and 6 (*right*), respectively. Group medians are connected by a *dashed line*, and the corresponding *t* test *P* values are displayed for each question. Question 4 reads “When you travel or leave home, do you sometimes forget to bring along your [health concern] medication(s)?” Question 6 reads “When you feel like your [health concern] is under control, do you sometimes stop taking your medication(s)?”

In general, there was no trend observed between the aggregate adherence metric and the observed total score on the MMAS8 (Fig. 6). However, upon inspection of responses to individual questions, a strong association was observed for one question, and one additional question showed a potential association. Question 4 on the MMAS8 showed a strong relationship in the log-domain and when tested using a non-parametric Kruskal–Wallis test in the linear domain (*P* = 0.011 and *P* = 0.02, respectively). Question 6 on the MMAS8 showed a potential association in the log-domain (*P* = 0.076). Figure 7 shows box plots for the distribution of calculated adherence metrics within each response group for questions 4 and 6; plots for all individual MMAS8 questions are provided as supplementary material. Additionally, despite the observed relationship with *ADHMET* values and gender, there was no correlation between gender and responses to questions 4 or 6 (see supplementary material).

## Discussion

In clinical practice, subjective interpretation of patient responses is often used as a means for decision-making. When such responses are intended to identify nonadherent behavior to a prescribed medication regiment, there is little evidence as to whether or not these responses will correlate with differences in objective measures of systemic exposure, which is ultimately the goal of treatment. This work was initiated to assess whether any such correlations exist between a particular clinical questionnaire designed to detect nonadherent behavior and expected plasma drug exposure levels in psychiatric patients on stable doses of aripiprazole. In other words, would results from a subjective questionnaire correlate with objective observations of steady-state plasma drug exposures in a representative patient population? To address this question, a novel “reverse-engineering”-based approach to sparse sampling population PK was applied.

From a pharmacometric standpoint, nonadherence to a prescribed medication may lead to decreased systemic exposure. However, nonadherence is bidirectional and can manifest as higher than intended systemic exposure when extra doses are taken. The clinical consequences and pharmacometric challenges of unobserved nonadherence has been discussed at large [1, 16, 18–20, 23, 24]. Despite this acknowledgment, little widespread utility has been found from using POPPK modeling as a tool for estimating adherence levels. Most efforts to mitigate nonadherence have focused on surrogate measures of compliance, such as electronic monitoring or patient journals [18, 19], while little effort has been made to quantify the degree to which nonadherence may be expected in a given population *a priori* under naturalistic conditions. The approach described in the current work provides a pharmacometric measure enabling the comparison of relative adherence across groups and, to our knowledge, has not been previously explored. Such a measure might be used in other areas during drug development; examples include using the *ADHMET* approach to distinguish between groups of responders and non-responders, or using observed *ADHMET* percentiles as a potential stratifier in a hazard function for a particular outcome.

The goal of establishing relative adherence levels to oral aripiprazole treatment post-approval allowed utility of the entire family of PK studies that were conducted during the approval process of this currently marketed drug. This is a unique use-case scenario for a pharmacometric analysis, given that most such analyses are performed to inform the approval process. We believe this approach offers a new avenue by which pharmacometricians and clinicians may collaborate to provide unique solutions throughout the entire lifespan of a compound. In this case, the lack of correlation of the aggregate adherence metric with the total score of the MMAS8 is noteworthy because study coordinators may use such questionnaires to set various inclusion criteria based on the implied inference of such a score. Although a formal validation of MMAS8 in psychiatric patients has not been conducted, the scale has previously been validated in patients with hypertension [25]. One possible reason for the lack of correlation of steady-state plasma drug concentrations with the MMAS8 total scores is patient bias in responses when answering clinical questionnaires [26, 27]. However, in the current study, we found that individual questions were more informative in certain cases than the overall questionnaire score. It was observed that MMAS8 question 4 “*When you travel or leave home, do you sometimes forget to bring along your [health concern] medication(s)?*” was a significant indicator (unadjusted *P* < 0.05) of the adherence metric in this data set: Patients who answered “yes” to this question had lower ADHMET values as a group than those who answered “no”. Although there was no correlation with total MMAS8 scores during a single visit investigation, the correlation of those test scores with *longer*-*term* behavioral tendencies assessed by PK sampling cannot be addressed by this work and still remains an unexplored topic. Given that screening, enrollment, and sampling were completed at the same visit, we believe that we captured the most accurate reflection of patient behavior with respect to plasma concentrations and willingness to answer the clinical questionnaire truthfully; however, it is also possible that patients may answer questionnaires more truthfully in a private session with their physician, leading to stronger correlations with medication exposure in practice.

A limitation of this method is the *a priori *need for a rich and robust data set that may be used for building a POPPK model capable of representing an upper bound of the true magnitude of variability associated with the model parameters. Such a data set may not exist during early clinical development, which may partially explain previous efforts to minimize the effect of nonadherence rather than to quantify it. However, it is possible that an *ADHMET* approach may be used after Phase 2a or in late clinical development (examples above) provided that adequate PK sampling had been conducted in earlier phases. It should be noted that this metric is cumulative and is not representative of adherence with the exact time of dosing; however, while the exact time of dosing becomes more critical as the dosing interval exceeds the terminal half-life of the compound, oral therapies are commonly developed for once-daily dosing accompanied by appreciable accumulation to steady-state, where minor deviations in the dosing time may not drastically affect the average profile. Another limitation of the work is its partial dependence on observed PK variability for a given compound, which could be a potential limiting factor for detecting relationships from a sample size perspective. Given that the aggregate adherence metric is a convolution of expected (normal) variability and adherence factors, it is not believed to be appropriate for indicating individual adherence levels—this is supported by our simplified simulation example. Additionally, because this study design dealt only with sparse sampling and a compound with modest variability in the clearance parameter (CV ~ 40 %), sensitivity and specificity at the individual level may change with a denser sampling strategy, lower variability in the model as a whole, or both. However, neither the magnitude of expected variability in the population, nor the lack of sensitivity at the individual level, are expected to effect the utility of the aggregate adherence metric for group comparisons—only the power to which a difference may be detected across those groups.

## Conclusions

A novel “reverse” approach to POPPK was applied as a primary endpoint in a clinical study to gauge the ultimate utility of a clinical questionnaire as a surrogate for medication exposure at steady-state. No observable relationship was found between the total score on the questionnaire (the MMAS8) and observed versus expected plasma concentrations of aripiprazole. However, because the clinical setting was a single-visit study of patients who were on stable doses of aripiprazole, extrapolation of these results to longer-term settings, or to correlations with responses provided to a physician in private, are not feasible. This application of POPPK may address a new type of clinical problem using pharmacometrics that is distinct from classic pharmacodynamic modeling.

## Electronic supplementary material

Below is the link to the electronic supplementary material.

Supplementary material 1 (DOCX 1574 kb)
