# Supplementary material for: Development and application of an aggregate adherence metric derived from population pharmacokinetics to inform clinical trial enrichment
Source: J Pharmacokinet Pharmacodyn. 2015 Mar 29;42(3):263–73. doi: 10.1007/s10928-015-9414-4 (PMC4432109; doi:10.1007/s10928-015-9414-4)
Supplement: Supplementary file 1 — Supplementary material 1 (DOCX 1574 kb) [file 10928_2015_9414_MOESM1_ESM.docx]

**Supplementary Material**

**Development and Application of an Aggregate Adherence Metric Derived from Population Pharmacokinetics to Inform Clinical Trial Enrichment**

Jonathan Knights, Shashank Rohatagi

**Example Simulation Control Stream**

$PROBLEM simulate adherence patterns

$INPUT C ID SIM ADH TRT DAY TIME AMT DV EVID CMT AGE SEX WT CYP2D6EM

$DATA ../ADH_NONMEM1.csv IGNORE=C

$SUBROUTINES ADVAN13 TRANS1 TOL=2 ;reduced TOL for simulation

$MODEL

; 2CM for parent

NCOMP=3

COMP = (GUT)

COMP = (VC_ARI)

COMP = (VP_ARI)

$PK

;NOTE THAT MU SAMPLING PRODUCES LOG VALUES OF THETAS THAT NEED TO BE EXPONENTIATED

IF (SEX.EQ.0) THEN

SEXO=1

ELSE

SEXO=0

ENDIF

;----------------------------------------------KA

TVKA=THETA(1) ;ABSORPTION CONSTANT rac3

LTVKA=LOG(TVKA)

MU_1 = LTVKA

KA = EXP(MU_1 + ETA(1))

;----------------------------------------------VC, VP, Q

;VOLUME OF CENTRAL COMPARTMENT

TVVC=THETA(2)

LTVVC=LOG(TVVC)

MU_2=LTVVC

VC = EXP(MU_2 + ETA(2))

;VOLUME OF PERIPHERAL COMPARTMENT

IF(WT.GT.0.AND.WT.LT.115) THEN

TVVP=(THETA(4) + THETA(6)*(WT-74.19) + THETA(7)*(AGE-32))*(1+SEXO*THETA(13))

ELSE

TVVP=(THETA(4) + THETA(7)*(AGE-32))*(1+SEXO*THETA(13))

ENDIF

LTVVP=LOG(TVVP)

MU_4=LTVVP

VP = EXP(MU_4 + ETA(4))

;Intercompartmental Transfer

IF (WT.GT.0.AND.WT.LT.115) THEN

WTOKQ=1

ELSE

WTOKQ=0

ENDIF

TVQA=THETA(3) + WTOKQ*THETA(9)*(WT-74.19) ;INTER-COMPARTMENTAL TRANSFER

TVQ=(TVQA)*(1+SEXO*THETA(12))

LTVQ=LOG(TVQ)

MU_3=LTVQ

Q = EXP(MU_3 + ETA(3))

;----------------------------------------------CLEARANCE (apparent) OF ARI (TOTAL)

IF (WT.GT.0.AND.WT.LT.115) THEN

WTPA=1

WTPB=0

ELSE IF (WT.GT.115) THEN

WTPA=0

WTPB=1

ELSE

WTPA=0

WTPB=0

ENDIF

IF (CYP2D6EM.EQ.0) THEN

PM=1

;missing values are -99

ELSE

PM=0

ENDIF

TVCLWT=THETA(5) + WTPA*THETA(8)*(WT - 74.19)

TVCLAGE=TVCLWT + THETA(10)*(AGE-32)

TVCL=TVCLAGE*(1+PM*THETA(11))

LTVCL=LOG(TVCL)

MU_5=LTVCL

CL = EXP(MU_5 + ETA(5))

;----------------------------------------------SCALING FACTORS

;scaling factors convert amount of drug in compartment to concentration

S2=VC/1000 ;C_UNITS IN NG/ML, DOSE IN MG

;----------------------------------------------DIFFERENTIAL EQS

$DES

DADT(1) = -KA*A(1)

DADT(2) = KA*A(1) - (CL/VC)*A(2) - (Q/VC)*A(2) + (Q/VP)*A(3)

DADT(3) = (Q/VC)*A(2) - (Q/VP)*A(3)

;----------------------------------------------RESIDUAL ERROR

$ERROR

IPRED=F

Y = IPRED*(1 + ERR(1)) ;proportional error model looked at first for both parent and metabolite

;----------------------------------------------INITIAL MODEL ESTIMATES

$THETA

(0.515) ;TH(1) KA(1/hr)

(192) ;TH(2) VC(L)

(12.2) ;TH(3) Q(L/hr)

(151) ;TH(4) VP(L)

(3.88) ;TH(5) CL(L/hr)

(4.07) ;TH(6) Linear relationship slope WT on VP

(0.915) ;TH(7) Linear AGE on VP

(0.0251) ;TH(8) linear relationship with weight on CL

(0.425) ;TH(9) Linear WT(+)<115 on Q

(-0.0167) ;TH(10) linear relationship with age on CL centered at 30

(-0.478) ;TH(11) Proportional shift in CL from CYP2D6 PM status

(0.543) ;TH(12) PROPORTIONAL(+) EFFECT OF SEX0 ON Q

(0.341) ;TH(13) PROPORTIONAL(+) EFFECT OF SEX0 ON VP

$OMEGA

0.398 ;ETA1 KA

$OMEGA BLOCK(3)

4.93E-02 ;ETA2 VC

3.33E-02 5.53E-02 ;ETA3 Q

4.59E-02 1.21E-02 7.67E-02 ;ETA4 VP

$OMEGA

0.153 ;ETA5 CL

$SIGMA

(0.0538) ;residual for parent

;———————————————————————SIMULATION AND TABLES

$SIMULATION (184158) ONLYSIMULATION

$TABLE NOPRINT FILE=ADH1.tab ONEHEADER ID ADH TRT AMT AGE SEX WT CYP2D6EM

TIME IPRED CWRESI CWRES NPDE KA CL VC VP

Q ETA1 ETA2 ETA3 ETA4 ETA5

**Example Estimation Control Stream**

$PROBLEM estimate parameters assuming full compliance

$INPUT C ID SIM CL_or ADH TRT DAY TIME AMT ADDL II DV EVID CMT AGE SEX WT CYP2D6EM CL_expect

$DATA ../ADH1_est.csv IGNORE=C

$SUBROUTINES ADVAN13 TRANS1 TOL=5

$MODEL

; 2CM for parent

NCOMP=3

COMP = (GUT)

COMP = (VC_ARI)

COMP = (VP_ARI)

$PK

;NOTE THAT MU SAMPLING PRODUCES LOG VALUES OF THETAS THAT NEED TO BE EXPONENTIATED

IF (SEX.EQ.0) THEN

SEXO=1

ELSE

SEXO=0

ENDIF

;----------------------------------------------KA

TVKA=THETA(1) ;ABSORPTION CONSTANT rac3

LTVKA=LOG(TVKA)

MU_1 = LTVKA

KA = EXP(MU_1 + ETA(1))

;----------------------------------------------VC, VP, Q

;VOLUME OF CENTRAL COMPARTMENT

TVVC=THETA(2)

LTVVC=LOG(TVVC)

MU_2=LTVVC

VC = EXP(MU_2 + ETA(2))

;VOLUME OF PERIPHERAL COMPARTMENT

IF(WT.GT.0.AND.WT.LT.115) THEN

TVVP=(THETA(4) + THETA(6)*(WT-74.19) + THETA(7)*(AGE-32))*(1+SEXO*THETA(13))

ELSE

TVVP=(THETA(4) + THETA(7)*(AGE-32))*(1+SEXO*THETA(13))

ENDIF

LTVVP=LOG(TVVP)

MU_4=LTVVP

VP = EXP(MU_4 + ETA(4))

;Intercompartmental Transfer

IF (WT.GT.0.AND.WT.LT.115) THEN

WTOKQ=1

ELSE

WTOKQ=0

ENDIF

TVQA=THETA(3) + WTOKQ*THETA(9)*(WT-74.19) ;INTER-COMPARTMENTAL TRANSFER

TVQ=(TVQA)*(1+SEXO*THETA(12))

LTVQ=LOG(TVQ)

MU_3=LTVQ

Q = EXP(MU_3 + ETA(3))

;----------------------------------------------CLEARANCE (apparent) OF ARI (TOTAL)

IF (WT.GT.0.AND.WT.LT.115) THEN

WTPA=1

WTPB=0

ELSE IF (WT.GT.115) THEN

WTPA=0

WTPB=1

ELSE

WTPA=0

WTPB=0

ENDIF

IF (CYP2D6EM.EQ.0) THEN

PM=1

;missing values are -99

ELSE

PM=0

ENDIF

TVCLWT=THETA(5) + WTPA*THETA(8)*(WT - 74.19)

TVCLAGE=TVCLWT + THETA(10)*(AGE-32)

TVCL=TVCLAGE*(1+PM*THETA(11))

LTVCL=LOG(TVCL)

MU_5=LTVCL

CL = EXP(MU_5 + ETA(5))

;----------------------------------------------SCALING FACTORS

;scaling factors convert amount of drug in compartment to concentration

S2=VC/1000 ;C_UNITS IN NG/ML, DOSE IN MG

;----------------------------------------------DIFFERENTIAL EQS

$DES

DADT(1) = -KA*A(1)

DADT(2) = KA*A(1) - (CL/VC)*A(2) - (Q/VC)*A(2) + (Q/VP)*A(3)

DADT(3) = (Q/VC)*A(2) - (Q/VP)*A(3)

;----------------------------------------------RESIDUAL ERROR

$ERROR

IPRED=F

Y = IPRED*(1 + ERR(1)) ;proportional error model looked at first for both parent and metabolite

;----------------------------------------------INITIAL MODEL ESTIMATES

$THETA

;all estimates from ******************.lst

(0.515 FIXED) ;TH(1) KA(1/hr) - 003_cov

(192 FIXED) ;TH(2) VC(L) - 007_cov

(12.2 FIXED) ;TH(3) Q(L/hr) - 007_cov

(151 FIXED) ;TH(4) VP(L) - 007_cov

(3.88 FIXED) ;TH(5) CL(L/hr) - 003_cov and 007_cov

(4.07 FIXED) ;TH(6) Linear relationship slope WT on VP

(0.915 FIXED) ;TH(7) Linear AGE on VP

(0.0251 FIXED) ;TH(8) linear relationship with weight on CL

(0.425 FIXED) ;TH(9) Linear WT(+)<115 on Q

(-0.0167 FIXED) ;TH(10) linear relationship with age on CL centered at 30

(-0.478 FIXED) ;TH(11) Proportional shift in CL from CYP2D6 PM status

(0.543 FIXED) ;TH(12) PROPORTIONAL(+) EFFECT OF SEX0 ON Q

(0.341 FIXED) ;TH(13) PROPORTIONAL(+) EFFECT OF SEX0 ON VP

$OMEGA

0.398 FIXED ;ETA1 KA

$OMEGA BLOCK(3) FIXED

4.93E-02 ;ETA2 VC

3.33E-02 5.53E-02 ;ETA3 Q

4.59E-02 1.21E-02 7.67E-02 ;ETA4 VP

$OMEGA

0.153 ;ETA5 CL

$SIGMA

(0.0538) ;residual for parent

;----------------------------------------------ESTIMATION METHODS AND TABLES

$EST METHOD=ITS INTERACTION FILE=ADH1_est.tbl NITER=100 PRINT=5 NOABORT SIGL=4 CITER=10

CALPHA=0.05 NOPRIOR=1

$EST METHOD=SAEM NBURN=500 ISAMPLE=200 NITER=500 PRINT=100

$EST METHOD=IMP EONLY=1 ISAMPLE=200 NITER=1000 MAPITER=0 PRINT=5 NOABORT CTYPE=3 SIGL=4 CITER=10

NOPRIOR=1 NSIG=3 MSFO=ADH1_est.msf

$COVARIANCE

$TABLE ID SIM ADH TRT TIME AMT DV EVID AGE SEX WT CYP2D6EM CL IPRED CL_or CL_expect ETA1 ETA2 ETA3 ETA4 ETA5 NOPRINT ONEHEADER FILE=ADH1_est.tab

**Adherence Metric Versus All MMAS8 Questions**

**VPCS: 24 Studies Used in Model Building (All Doses)**


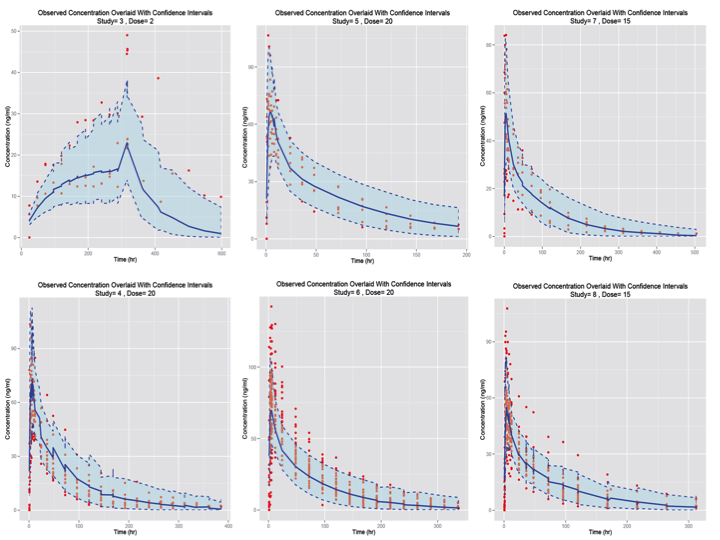


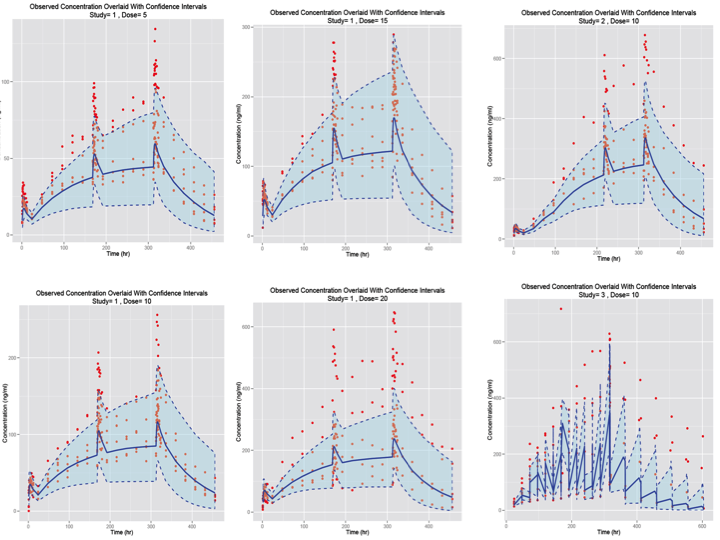


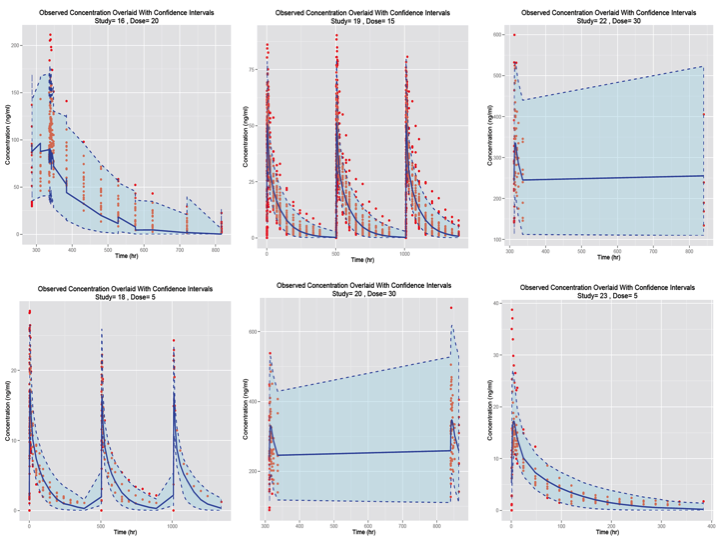

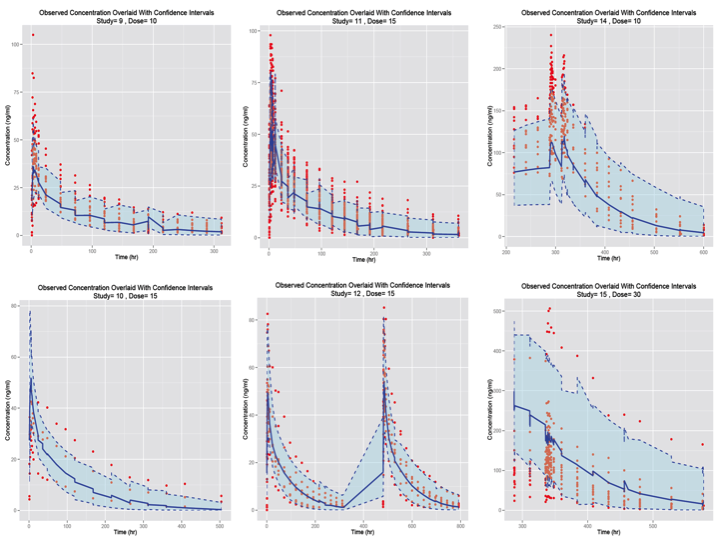


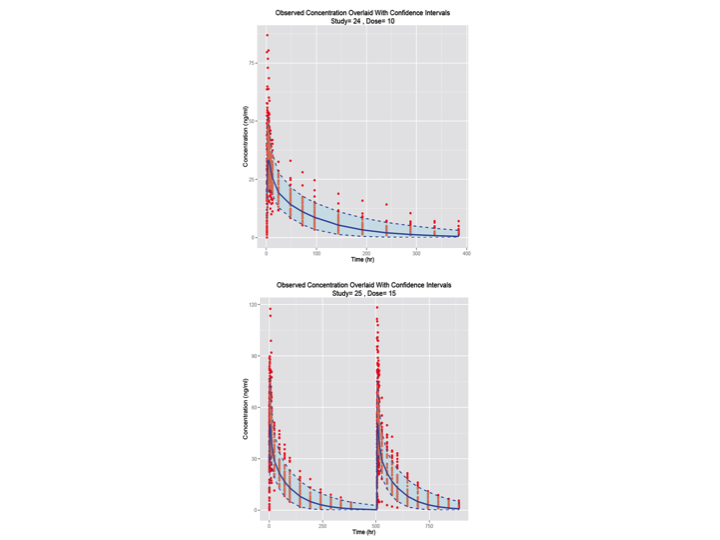


**Aggregate Adherence Metric vs. Gender**

| Gender ~ MMAS04 | | |
| --- | --- | --- |
| SEX | MMAS04 | N |
| 0 | 0 | 9 |
| 0 | 1 | 7 |
| 1 | 0 | 22 |
| 1 | 1 | 9 |
|  |  |  |
| chi square with Yate's continuity | | |
|  | p-value = | 0.4939 |
|  |  |  |
| Gender ~ MMAS06 | | |
| SEX | MMAS06 | N |
| 0 | 0 | 8 |
| 0 | 1 | 8 |
| 1 | 0 | 13 |
| 1 | 1 | 18 |
|  |  |  |
| chi square with Yate's continuity | | |
|  | p-value = | 0.8279 |

**References**

1. Velligan DI, Weiden PJ, Sajatovic M, Scott J, Carpenter D, Ross D, Docherty JP (2010) Strategies for addressing adherence problems in patients with serious and persistent mental illness: recommendations from the expert consensus guidelines. J Psychiatr Pract 16(5):306-324
